# Supplementary material for: Profiling Analysis of Tryptophan Metabolites in the Urine of Patients with Parkinson’s Disease Using LC–MS/MS
Source: Pharmaceuticals (Basel). 2023 Oct 20;16(10):1495. doi: 10.3390/ph16101495 (PMC10610059; doi:10.3390/ph16101495)
Supplement: Supplementary file 1 [file pharmaceuticals-16-01495-s001.zip › pharmaceuticals-2650700-supplementary.pdf]

Fig. S1. Influences of mobile phase additives: (a) 0.1% formic acid, (b) 0.1% trifluoroacetic acid, and (c) 0.1% heptafluorobutyric acid

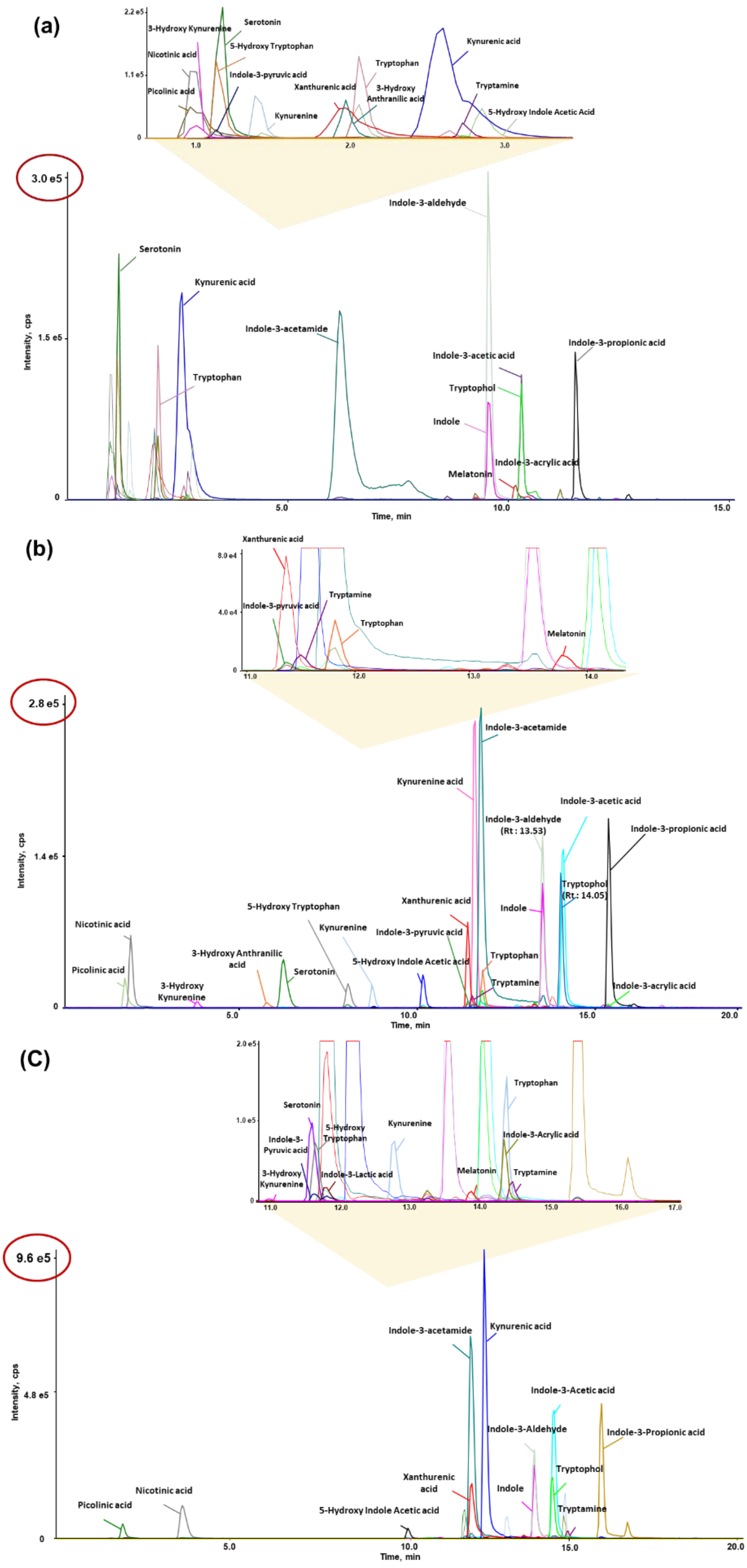

Fig. S2. Overlaid MRM chromatograms of standard mixture of 21 tryptophan metabolites.

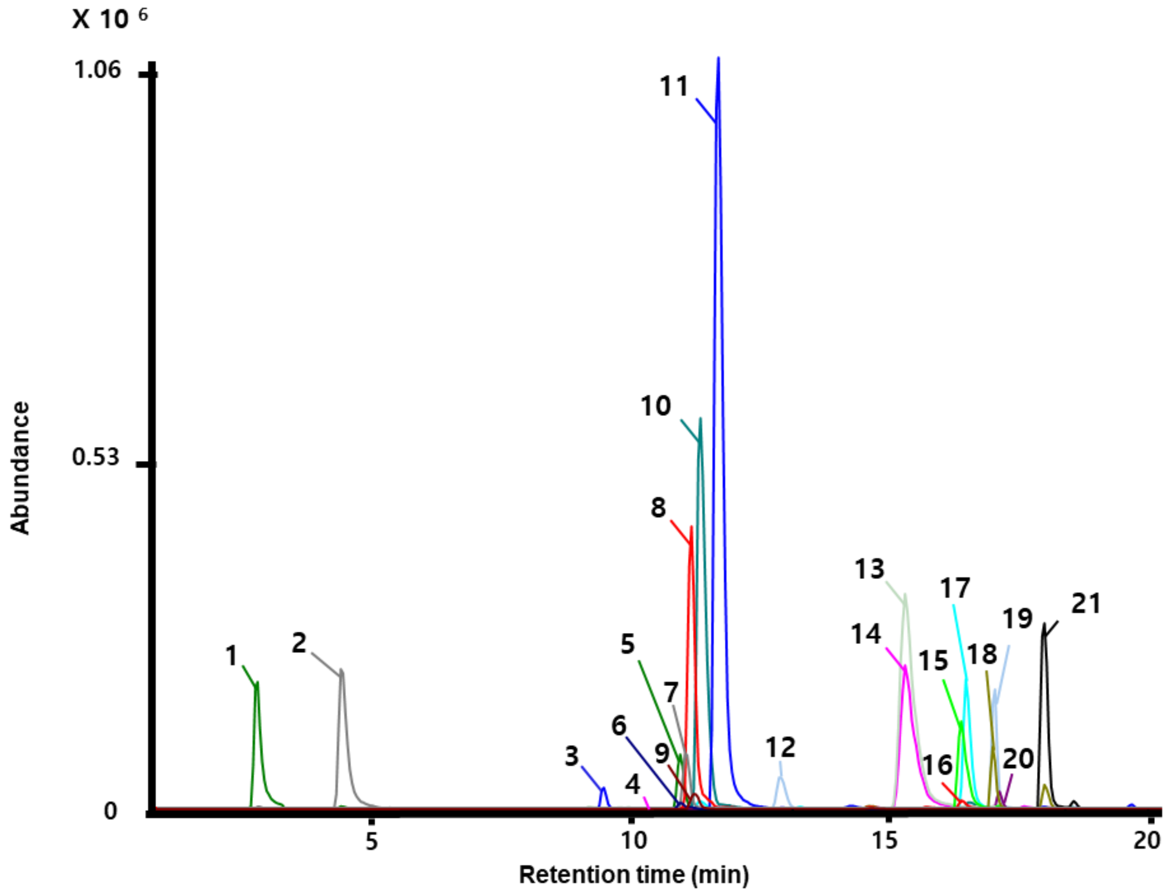

* The peaks were identified as follows; 1. picolinic acid, 2. nicotinic acid, 3. 5-hydroxyl indole acetic acid, 4. 3-hydroxy kynurenine, 5. serotonin, 6. indole-3-pyruvic acid, 7. 5-hydroxy tryptophan, 8. xanthurenic acid, 9. indole-3-lactic acid, 10. indole-3-acetamide, 11. kynurenic acid, 12. kynurenine, 13. indole-3-aldehyde, 14. indole, 15. tryptophol, 16. melatonin, 17. indole-3-acetic acid, 18. indole-3-acrylic acid, 19. tryptophan, 20. tryptamine, 21. indole-3-propionic acid

Fig. S3. Overlaid MRM chromatograms of 21 tryptophan metabolites in human urine sample.

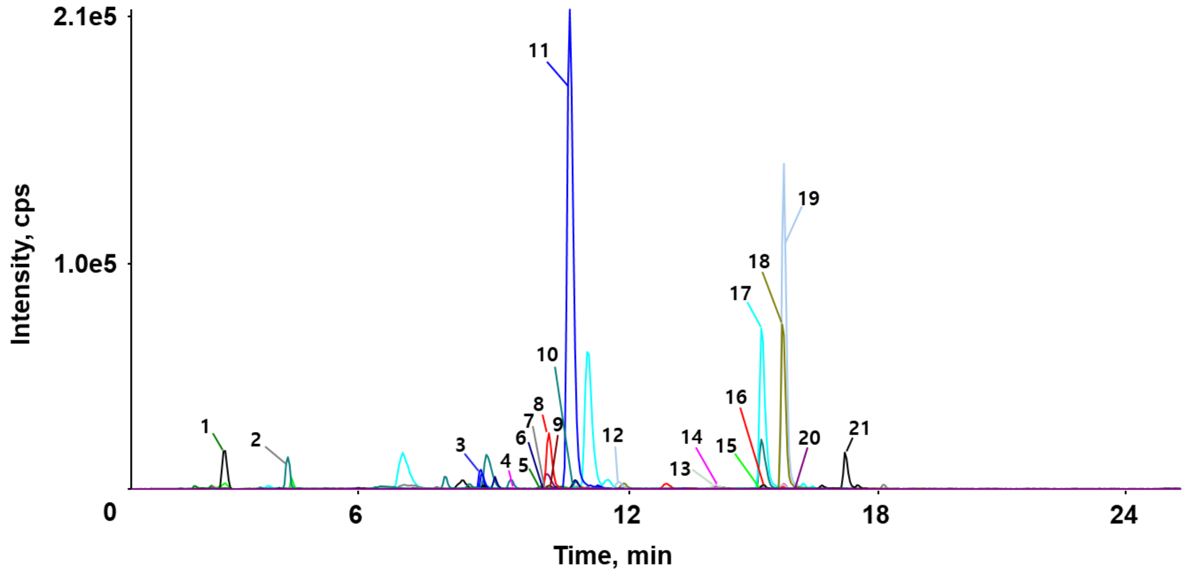

* The peaks were identified as follows; 1. picolinic acid, 2. nicotinic acid, 3. 5-hydroxyl indole acetic acid, 4. 3-hydroxy kynurenine, 5. serotonin, 6. indole-3-pyruvic acid, 7. 5-hydroxy tryptophan, 8. xanthurenic acid, 9. indole-3-lactic acid, 10. indole-3-acetamide, 11. kynurenic acid, 12. kynurenine, 13. indole-3-aldehyde, 14. indole, 15. tryptophol, 16. melatonin, 17. indole-3-acetic acid, 18. indole-3-acrylic acid, 19. tryptophan, 20. tryptamine, 21. indole-3-propionic acid

Fig. S4. Heat-map analysis for 21 tryptophan metabolites in urine samples.

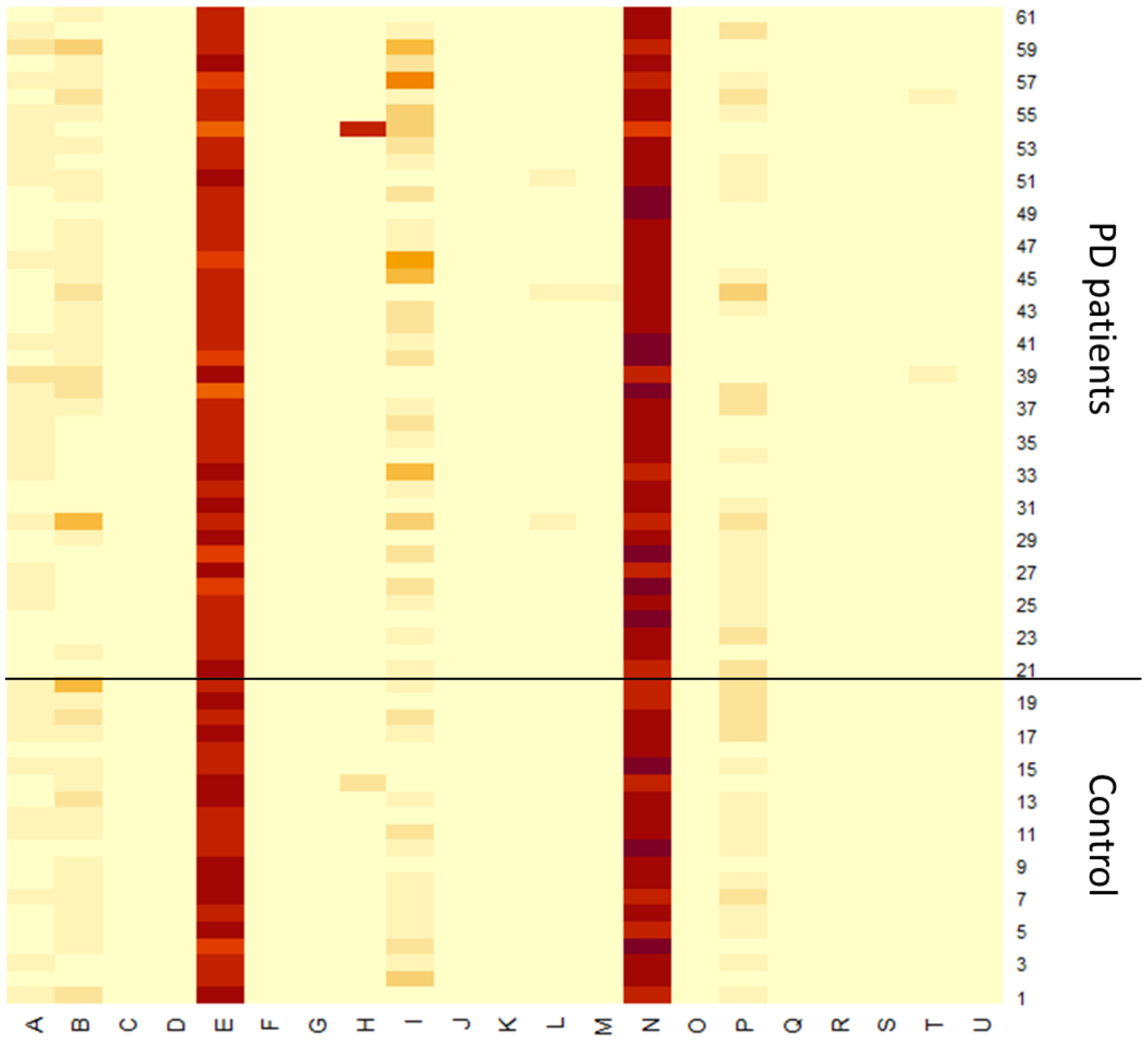

* The alphabetics were identified as follows; A. 5-hydroxyl indole acetic acid, B. melatonin, C. serotonin, D. 5-hydroxy tryptophan, E. tryptophan, F. indole, G. indole-3-acetamide, H. tryptamine, I. indole-3-acetic acid, J. indole-3-aldehyde, K. tryptophol, L. indole-3-pyruvic acid, M. indole-3-lactic acid, N. indole-3-acrylic acid, O. indole-3-propionic acid, P. kynurenic acid, Q. xanthurenic acid, R. picolinic acid, S. nicotinic acid, T. kynurenine, U. 3-hydroxy kynurenine.

Fig. S5. Heat-map for stability and matrix effect of 21 tryptophan metabolites.

Analytes	Conc. (ng/mL)	Stability (%)							Matrix effect (%)
		room temperature			4 °C (autosampler)			3 Freeze-thaw cycle	
		24 hr	48 hr	72 hr	24 hr	48 hr	72 hr		
5-Hydroxyl Indole Acetic Acid	150								
	5,000								
	10,000								
Melatonin	150								
	5,000								
	10,000								
Serotonin	150								
	1,000								
	2,000								
5-Hydroxy Tryptophan	150								
	500								
	1,000								
Tryptophan	10,000								
	15,000								
	20,000								
Indole	150								
	1,000								
	2,000								
Indole-3-Acetamide	150								
	500								
	1,000								
Tryptamine	150								
	500								
	1,000								
Indole-3-Acetic Acid	150								
	1,000								
	2,000								
Indole-3-Aldehyde	150								
	1,000								
	2,000								
Tryptophol	150								
	500								
	1,000								
Indole-3-Pyruvic Acid	150								
	5,000								
	10,000								
Indole-3-Lactic Acid	150								
	1,000								
	2,000								
Indole Acrylic Acid	10,000								
	15,000								
	20,000								
Indole-3-Propionic Acid	150								
	1,000								
	2,000								
Kynurenic Acid	150								
	5,000								
	10,000								
Xanthurenic acid	150								
	500								
	1,000								
Picolinic acid	150								
	500								
	1,000								
Nicotinic acid	150								
	500								
	1,000								
Kynurenine	150								
	5,000								
	10,000								
3-Hydroxy Kynurenine	150								
	500								
	1,000								

Table S1. Analytical characteristics of the established method for biological samples.

Analytes	Calibration range (ng/mL)	Linear regression equation	R^2	LODs (ng/mL)	LOQs (ng/mL)	Spiked Concentration (ng/mL)	Intra-day assay (%)	Inter-day assay (%)	Recovery (%)
5-Hydroxyl Indole Acetic Acid	150-10000	y = 0.0182x + 0.8708	0.997	5	20	150	107.9 (4.7)	113.0 (14.3)	87.8
						5,000	97.9 (1.8)	98.3 (5.3)	103.2
						10,000	87.6 (6.3)	87.0 (3.9)	85.8
Melatonin	150-10000	y = 0.0141x + 1.4036	0.998	25	75	150	110.2 (7.5)	113.8 (12.9)	91.1
						5,000	89.2 (1.0)	89.7 (5.2)	99.9
						10,000	87.3 (3.3)	85.5 (4.5)	94.4
Serotonin	150-2000	y = 0.0376x + 1.154	0.998	5	20	150	96.7 (4.9)	92.2 (13.7)	108.0
						1,000	90.0 (8.9)	93.6 (13.9)	99.1
						2,000	95.8 (7.1)	96.0 (6.7)	105.0
5-Hydroxy Tryptophan	150-1000	y = 0.0385x + 1.2631	0.996	25	75	150	97.4 (3.3)	99.6 (9.6)	87.5
						500	88.1 (6.6)	86.2 (6.8)	109.9
						1,000	100.5 (9.3)	114.1 (11.6)	97.8
Tryptophan	10000-20000	y = 0.0558x + 1.8818	0.999	1	3	10,000	96.5 (3.2)	90.5 (5.9)	93.6
						15,000	106.9 (12.8)	112.7 (11.8)	91.4
						20,000	113.5 (4.1)	114.4 (9.1)	94.9
Indole	150-2000	y = 0.0823x + 6.4491	0.998	1	5	150	105.7 (3.3)	114.3 (7.1)	89.6
						1,000	113.3 (9.0)	111.7 (9.0)	108.9
						2,000	89.8 (9.8)	97.6 (13.2)	89.4
Indole-3-Acetamide	150-1000	y = 0.0719x + 28.566	0.991	1	5	150	108.0 (6.4)	114.1 (11.2)	82.5
						500	110.1 (12.2)	103.5 (14.3)	86.8
						1,000	95.7 (1.5)	95.7 (1.5)	83.7

Tryptamine	150-1000	y = 0.0049x + 1.5384	0.999	25	100	150	98.7 (4.0)	109.0 (12.0)	94.7
						500	98.7 (11.5)	99.4 (8.1)	90.9
						1,000	101.7 (1.8)	102.9 (5.6)	86.3
Indole-3-Acetic Acid	150-2000	y = 0.0554x + 28.651	0.991	1	5	150	101.6 (1.6)	103.5 (12.6)	93.1
						1,000	103.7 (6.0)	101.7 (6.2)	96.7
						2,000	94.5 (12.8)	102.9 (13.4)	90.2
Indole-3-Aldehyde	150-2000	y = 0.0562x + 5.8258	0.999	25	100	150	109.7 (2.8)	110.5 (12.7)	94.6
						1,000	108.8 (8.9)	106.0 (7.1)	85.3
						2,000	93.9 (10.7)	102.2 (13.3)	90.7
Tryptophol	150-1000	y = 0.0582x + 1.843	0.990	5	10	150	100.5 (10.6)	114.1 (10.6)	97.5
						500	111.6 (3.8)	114.0 (5.5)	104.6
						1,000	95.8 (8.0)	92.7 (6.5)	89.5
Indole-3-Pyruvic Acid	150-10000	y = 0.0086x - 0.0033	0.996	50	150	150	110.7 (11.5)	113.2 (11.1)	92.9
						5,000	89.1 (7.3)	90.7 (12.9)	87.9
						10,000	97.1 (3.2)	89.6 (11.6)	94.3
Indole-3-Lactic Acid	150-2000	y = 0.0039x + 0.2053	0.996	1	150	150	88.0 (10.6)	86.2 (6.0)	97.1
						1,000	93.9 (10.4)	92.0 (6.7)	92.6
						2,000	91.7 (9.3)	90.1 (13.2)	97.0
Indole Acrylic Acid	10000-20000	y = 0.0094x + 0.9321	0.998	1	5	10,000	100.9 (13.8)	102.6 (12.8)	102.6
						15,000	89.3 (4.3)	85.9 (13.0)	103.1
						20,000	94.0 (5.0)	86.0 (6.4)	84.1
Indole-3-Propionic Acid	150-2000	y = 0.0627x + 5.0551	0.995	1	5	150	110.1 (11.4)	111.8 (14.1)	91.8
						1,000	99.8 (2.7)	106.4 (7.1)	98.8
						2,000	103.6 (2.5)	98.9 (14.3)	89.1
Kynurenic Acid	150-10000	y = 0.134x + 1.677	0.995	1	5	150	99.9 (11.8)	100.2 (11.6)	88.4
						5,000	108.5 (2.0)	104.3 (5.7)	89.1
						10,000	92.5 (7.1)	86.9 (10.2)	84.7
Xanthurenic acid	150-1000	y = 0.1819x - 7.9971	0.997	1	3	150	88.0 (10.0)	88.8 (6.2)	104.8
						500	107.6 (13.0)	102.2 (9.3)	101.3
						1,000	111.0 (13.8)	107.2 (13.7)	87.9

Picolinic acid	150-1000	y = 0.0184x - 2.1014	0.991	50	75	150	113.2 (3.3)	114.4 (3.9)	100.0
						500	89.5 (13.3)	94.8 (12.9)	89.6
						1,000	96.0 (6.2)	103.9 (10.6)	84.6
Nicotinic acid	150-1000	y = 0.009x - 0.2465	0.994	150	300	150	107.5 (8.9)	112.0 (9.7)	113.2
						500	102.6 (1.4)	105.3 (12.2)	94.6
						1,000	112.0 (1.8)	109.3 (13.7)	87.5
Kynurenine	150-10000	y = 0.0263x + 0.7919	0.992	10	20	150	86.3 (4.8)	86.9 (4.3)	93.4
						5,000	104.9 (7.3)	98.4 (10.5)	86.9
						10,000	100.9 (0.8)	101.1 (3.8)	99.5
3-Hydroxy Kynurenine	150-1000	y = 0.0029x - 0.2752	0.991	150	300	150	94.2 (9.6)	86.6 (12.6)	82.7
						500	109.4 (3.2)	112.2 (11.8)	82.8
						1,000	86.1 (1.9)	93.4 (13.5)	82.5

Table S2. Stability and matrix effect of tryptophan metabolites in spiked urine sample.

Analytes	Conc. (ng/mL)	Stability (%)							Matrix effect (%)
		room temperature			4 °C (autosampler)			3 Freeze-thaw cycle	
		24 hr	48 hr	72 hr	24 hr	48 hr	72 hr		
5-Hydroxyl Indole Acetic Acid	150	108.1	104.7	97.3	97.5	95.5	87.1	104.5	104.3
	5,000	101.4	97.6	98.1	97.7	101.6	103.1	108.8	
	10,000	92.4	92.4	98.1	100.7	94.5	100.2	97.6	
Melatonin	150	105.4	103.2	97.7	99.7	97.7	86.8	105.0	95.9
	5,000	103.0	101.6	103.1	97.7	101.6	103.1	98.3	
	10,000	86.5	82.0	90.3	97.4	98.2	101.2	94.8	
Serotonin	150	96.4	84.5	84.7	84.1	87.2	91.7	87.7	101.3
	1,000	80.5	109.2	96.8	103.7	99.1	101.9	95.7	
	2,000	102.2	101.8	100.5	98.4	98.0	102.3	100.0	
5-Hydroxy Tryptophan	150	87.5	85.8	81.5	91.5	87.0	89.8	84.3	95.3
	500	89.1	91.2	88.6	93.8	98.8	103.2	112.4	
	1,000	90.8	90.8	73.4	89.8	101.9	93.6	117.1	
Tryptophan	10,000	101.1	98.7	100.1	101.1	98.7	102.7	104.7	95.3
	15,000	94.2	102.8	99.0	99.0	101.5	100.6	114.6	
	20,000	86.2	88.5	90.4	98.5	97.8	93.3	115.7	
Indole	150	103.6	90.4	90.0	95.4	104.7	98.6	104.3	98.5
	1,000	90.2	82.9	83.9	90.8	98.8	94.7	85.1	
	2,000	94.6	99.6	88.1	88.1	93.1	97.9	102.6	
Indole-3-Acetamide	150	81.6	103.9	95.5	97.9	101.8	97.2	112.4	86.1
	500	97.5	100.0	100.0	97.9	80.2	81.2	85.4	
	1,000	103.3	101.3	91.7	94.2	88.5	97.3	113.0	

	150	86.4	83.6	84.4	100.7	87.8	95.3	90.1	
Tryptamine	500	96.5	93.4	78.4	86.7	96.2	83.8	96.4	96.5
	1,000	102.0	87.2	89.3	95.7	97.8	97.6	92.0	
	150	89.7	91.6	101.2	99.3	99.2	98.2	101.5	
Indole-3-Acetic Acid	1,000	102.8	102.9	88.5	96.6	102.3	102.3	109.9	96.1
	2,000	91.1	101.4	82.9	91.1	91.5	97.8	97.3	
	150	97.6	87.7	92.6	99.2	93.2	88.8	99.4	
Indole-3-Aldehyde	1,000	92.1	95.0	86.8	100.2	97.1	99.7	103.7	92.1
	2,000	89.7	99.0	83.6	89.7	99.2	96.7	103.8	
	150	100.1	101.9	93.5	101.5	103.5	87.7	102.0	
Tryptophol	500	90.9	88.7	79.8	92.5	98.9	92.7	92.9	94.1
	1,000	102.8	100.1	89.4	99.4	99.1	97.1	98.5	
	150	102.1	98.4	94.7	101.4	89.4	83.8	100.1	
Indole-3-Pyruvic Acid	5,000	92.4	99.0	85.8	92.4	86.1	93.3	97.6	102.2
	10,000	97.7	93.3	91.7	102.1	91.0	95.7	69.1	
	150	94.2	92.3	97.1	97.8	98.8	102.2	106.2	
Indole-3-Lactic Acid	1,000	99.6	101.6	78.2	96.3	95.5	99.9	101.9	91.8
	2,000	93.1	78.0	82.3	88.7	100.4	87.2	106.0	
	10,000	99.4	99.6	98.3	91.1	103.0	99.5	105.9	
Indole Acrylic Acid	15,000	89.3	78.5	79.7	99.3	95.9	99.9	110.8	98.2
	20,000	91.7	78.1	83.6	101.9	100.8	95.2	105.3	
Indole-3-	150	87.7	105.7	91.6	99.4	101.6	96.9	111.4	
Propionic	1,000	97.2	96.2	93.5	101.0	98.4	98.1	96.5	95.6
Acid	2,000	98.3	93.9	83.4	91.9	94.7	99.3	96.9	
	150	101.3	100.7	85.6	91.6	90.7	81.5	97.0	
Kynurenic Acid	5,000	103.0	98.9	94.7	98.2	99.3	99.5	101.5	93.1
	10,000	106.5	99.2	93.4	95.0	89.0	87.4	76.3	
	150	95.5	92.5	84.2	97.4	105.1	104.1	98.0	
Xanthurenic acid	500	93.7	82.6	74.8	99.1	102.3	103.0	112.4	85.5
	1,000	92.1	76.7	91.3	81.2	102.6	101.5	111.6	
Picolinic acid	150	92.2	90.9	88.2	102.6	101.6	98.1	101.9	97.6
	500	99.9	98.2	91.6	97.8	102.8	98.3	101.6	

	1,000	102.6	95.6	84.9	99.2	95.6	95.2	111.3	
Nicotinic acid	150	82.5	98.8	81.5	96.3	103.9	99.4	95.5	
	500	90.6	95.8	96.8	100.2	97.8	101.0	102.0	96.0
	1,000	102.5	101.7	89.6	94.1	80.6	88.8	115.5	
Kynurenine	150	89.2	85.8	82.0	100.0	101.8	94.5	101.7	
	5,000	92.9	98.8	89.7	92.9	103.9	103.8	113.7	93.2
	10,000	101.3	73.3	73.8	97.4	99.6	87.9	103.4	
3-Hydroxy Kynurenine	150	93.4	92.7	86.0	89.5	92.7	85.8	94.1	
	500	101.0	89.7	86.9	100.0	103.3	86.6	110.5	87.5
	1,000	99.7	95.3	92.6	96.3	92.7	87.3	93.1	

Table S3. Information for collected urine samples.

Samples		Control group	PD patients
Sex	Male	13	16
	Female	7	25
Age		69.69±6.76	65.05±7.76

Table S4. MS parameters for multiple reaction monitoring mode.

Compound name	Declustering Potential (DP)	Entrance Potential (EP)	Quantifier ion transition	Collision Energy	Qualifier ion transition	Collision Energy
5-Hydroxyl indole acetic acid	30	4	192 > 146	30	192 > 119	30
Melatonin	40	7	233 > 173	24	233 > 159	24
Serotonin	30	4	177 > 160	20	177 > 132	20
5-Hydroxy tryptophan	30	5	221 > 161	13	221 > 162	22
Tryptophan	40	5	205 > 188	20	205 > 146	25
Indole	40	7	118 > 91	38	118 > 65	41
Indole-3-acetamide	30	6	175 > 130	30	175 > 158	24
Tryptamine	20	5	161 > 143	16	161 > 117	21
Indole-3-acetic acid	40	7	176 > 130	24	176 > 103	29
Indole-3-aldehyde	40	4	146 > 118	23	146 > 91	31
Tryptophol	30	5	162 > 90	15	162 > 117	28
Indole-3-pyruvic acid	40	9	204 > 158	27	204 > 130	46
Indole-3-lactic acid	70	12	206 > 143	16	206 > 130	65
Indole-3-acylic acid	50	7	188 > 115	70	188 > 170	22
Indole-3-propionic acid	30	7	190 > 130	19	190 > 79	26
Kynurenic acid	30	9	190 > 144	30	190 > 162	28

Xanthurenic acid	40	6	206 > 160	33	206 > 132	34
Picolinic acid	30	5	124 > 78	14	124 > 78	31
Nicotinic acid	40	6	124 > 78	32	124 > 52	43
Kynurenine	30	7	209 > 94	23	209 > 146	22
3-Hydroxy kynurenine	30	5	225 > 110	20	225 > 161	22
